# Papillon-Lefévre Syndrome: A Rare Case Report and a Brief Review of Literature

**DOI:** 10.7759/cureus.26163

**Published:** 2022-06-21

**Authors:** Nishath Sayed Abdul, Lamis Khalid Dagriri, Mahesh Shenoy

**Affiliations:** 1 Oral and Maxillofacial Surgery and Diagnostic Sciences, College of Dentistry Riyadh Elm University, Riyadh, SAU; 2 Dentistry, College of Dentistry Riyadh Elm University, Riyadh, SAU

**Keywords:** saudi arabia, consanguinity, periodontitis, palmar- plantar hyperkeratosis, papillon-lefevre syndrome

## Abstract

Background: Palmar-plantar hyperkeratosis and severe early-onset periodontitis are the hallmarks of the uncommon autosomal recessive Papillon-Lefévre syndrome (PLS), which may cause both primary and permanent teeth to be lost at an early age. The cause and pathophysiology of the disorder involve several factors, including genetic, immunological, and microbial factors.

Aim: The purpose of this case study is to provide insight into the fascinating role of consanguinity in the aetiology of this unusual illness.

Case Presentation: An unusual PLS case report in a household with two consanguineously married parents was provided. A 17-year-old Saudi boy visited the dental clinic at Riyadh Elm University because he was having problems with loose teeth and pain while chewing, as well as irritated and friable gums. He may be suffering from a genetic condition that has been effectively treated in the past by his elder brother, who is now 26 years old. In this instance, severe extensive periodontitis contributed to the early loss of primary teeth as well as permanent teeth, resulting in PLS. On the lateral surface of the soles, the distinctive skin lesions revealed hyperkeratosis with regions of persistent thickening, flaking, and scaling. There were erythematous patches on the palms, but no hyperkeratosis was seen.

Conclusion: When it comes to Papillon-Lefévre syndrome (PLS), this is an extremely unusual instance since two siblings in the same family were both afflicted. Patients who are stigmatised because of their condition will benefit from early discovery and multidisciplinary treatment.

## Introduction

The Papillon-Lefévre condition (PLS) palmoplantar keratosis type IV is an extraordinary autosomal latent skin disorder (PPK). M.M. Papillon and Paul Lefévre were two French professionals who gave it these names in the mid-1920s [1]. The premature loss of crucial and long-lasting teeth [2-5] was caused by hyperkeratosis (palmar-plantar) and a significant solid start to periodontitis (periodontitis) [6]. PLS affects 1-4 people out of every million people worldwide. Autosomal recessive inheritance and the presence of PPK are diagnostic criteria for PLS. There are two key hallmarks of the PLS disorder: severe periodontal disease and palmoplantar keratosis. Recently, research has shown a correlation between the prevalence of PLS in families with close genetic ties, as well as in particular ethnic groups. One study found that 75% of Arabs were descended from parents with whom they had a close relationship [6]. PLS was shown to be more prevalent among Arab and Indian people, as well as isolated communities [7-11]. Children with PLS may have long-term functional, esthetic, psychological, and social consequences due to the rapid deterioration of their oral and skin alterations throughout their formative years. Presented here is a case of PLS in a 17-year-old Saudi male from Milan. The patient gave permission for the procedure to go forward. The University Research Centre and the institution Review Board authorized the case number FRP/2021/432/710/685. This case was treated according to the Declaration of Helsinki issued by the World Medical Association.

## Case presentation

Dentists at Riyadh Elm University in Saudi Arabia saw a 17-year-old male patient who had a primary complaint of tooth mobility and the need to replace missing teeth. Proper informed consent was taken from the patient, and also an ethical approval letter was taken from Institutional review board of Riyadh Elm University with a number FRP/2021/432/710/685. Photographic consent was also taken from the patient for the same. His family history revealed that his parents were first cousins and had a consanguineous marriage, which may have contributed to his condition. His parents were not affected and had an uneventful pregnancy and delivery. The patient’s elder brother, aged 26 years, showed a similar condition at eight years of age and was treated successfully a few years ago.

The onset of this condition was at four years of age for the patient, as revealed by his parents, and he did not seek any treatment during that period. The symptoms are aggravated during the cold winter season, making it difficult to walk. His nails and hair seemed normal. The patient was sweating profusely.

When he was three years old, he started losing his main teeth, and by the time he was five or six years old, he had lost all of them. However, because of their inherent brittleness, the bulk of permanent teeth is lost through a series of exfoliations rather than all at once. Because of the early loss of primary and permanent teeth, chewing was difficult. There was no medical history that influenced the outcome.

Clinical examination

**Figure 1:**
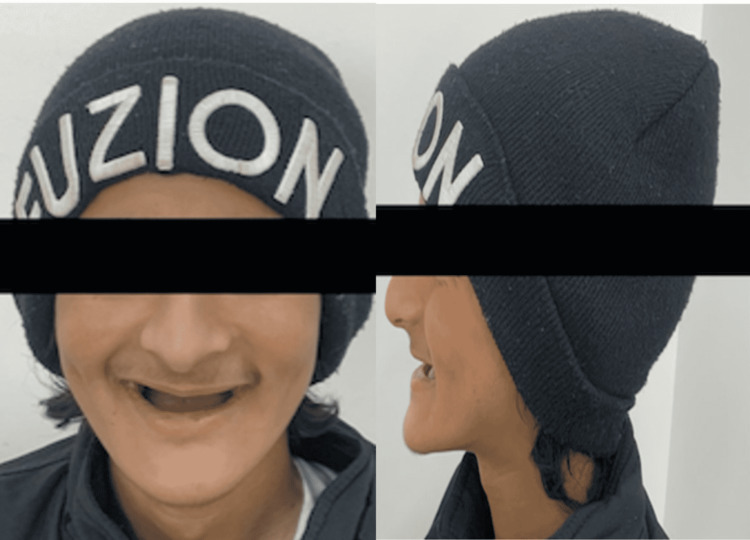
Showing frontal and lateral profile of the patient face reveals a loss of vertical facial height and edentulous appearance due to early loss of teeth.

After a general evaluation, it was found that both the patient's physical and mental growth were normal. A checkup outside of the mouth showed that tooth loss had caused the vertical height of the face to reduce (Figure 1).

**Figure 2:**
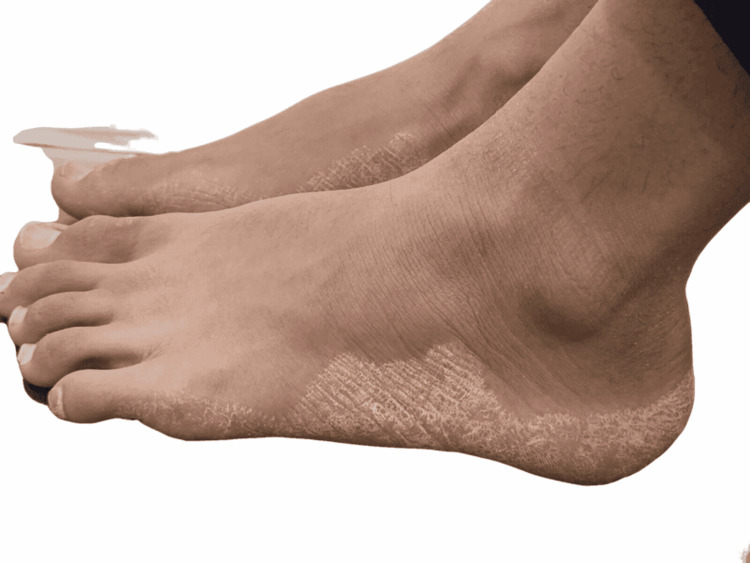
Examination of soles revealed palmoplantar keratosis appearing as symmetric, well-demarcated, scaly, keratotic plaques over the skin of his soles

Dermatological examination revealed the presence of symmetric, well-demarcated, white, rough, scaly hyperkeratotic confluent plaques affecting the plantar surface of his soles (Figure 2).

**Figure 3:**
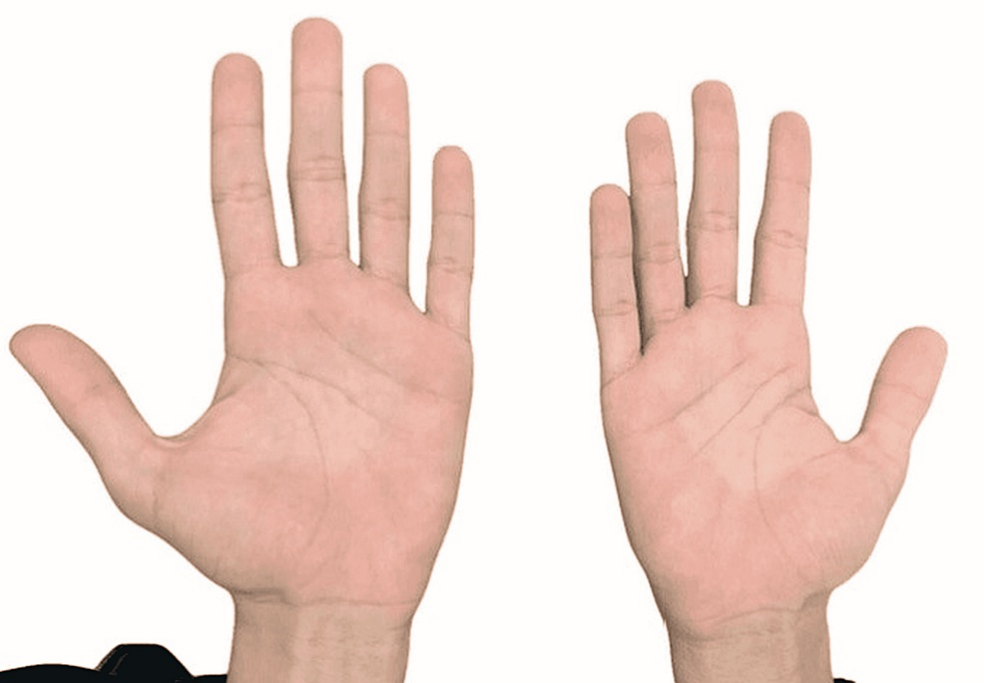
Areas of erythema on the palms were seen

The hands were swollen and red in spots. The palms, knees, and elbows, on the other hand, were clear of keratosis (Figure 3).

**Figure 4:**
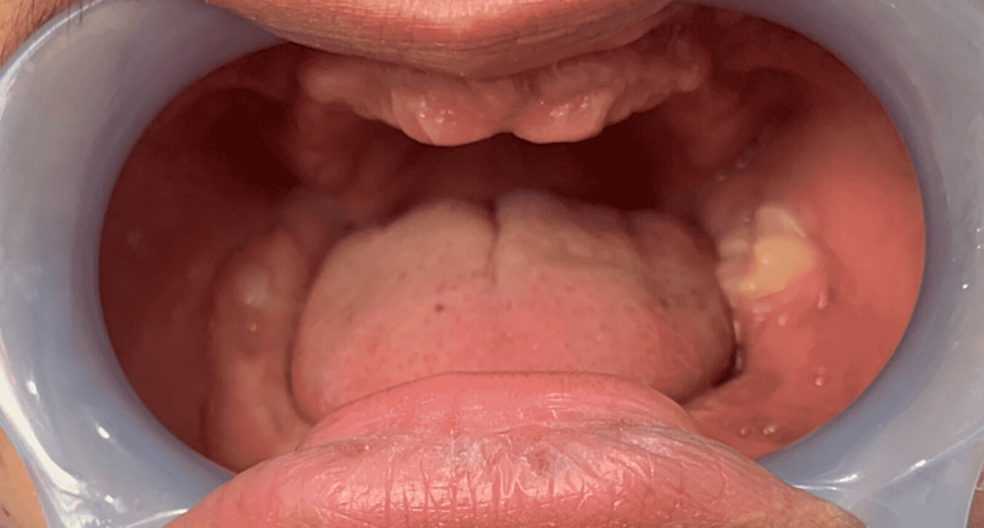
Mobile teeth were extracted. Generalized Loss of alveolar bone was evident

Intra-oral examination revealed the presence of mobile permanent maxillary left canine, mandibular right canine and mandibular right third molar, left second molar before extraction. Some of these teeth had red and swollen gums that were also showing signs of gingival recession. The edentulous portions have normal mucosa. Offensive oral malodor was present. Extraction of the mobile teeth was done as shown in Figure 4.

Radiographic examination

**Figure 5:**
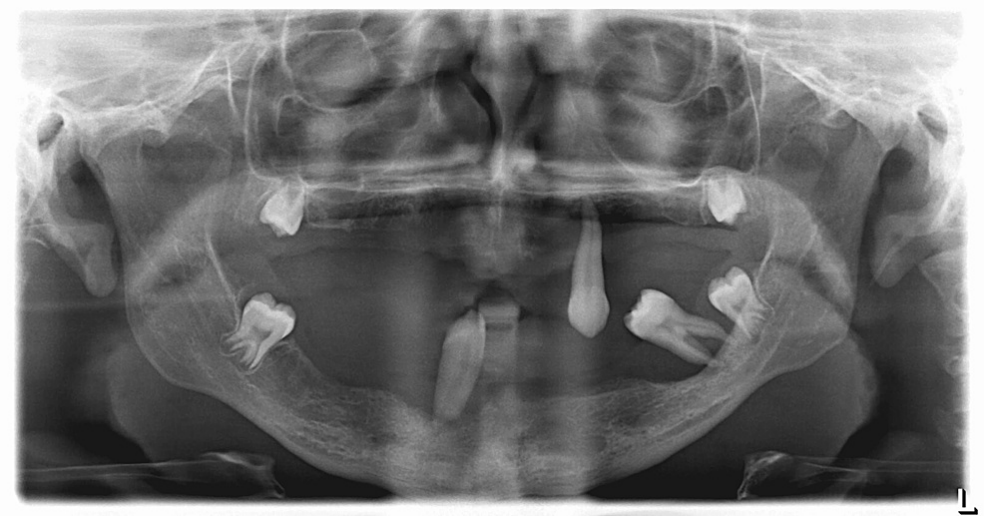
A panoramic scan showed several missing teeth, extensive interdental alveolar bone loss, and teeth that seemed to be "floating in air" due to their increased spacing and gingival recession up to the apical third of roots on the patient.

A panoramic radiograph showed the presence of maxillary third molars and mandibular left third molar, which were not evident clinically. Generalized alveolar bone loss floating in the air-like appearance of remaining teeth was evident. A decreased vertical facial height was noted. The lateral skull radiograph showed no evidence of intracranial calcifications (Figure 5).

Hematological and biochemical tests were performed in the laboratory. The results of routine blood tests and liver function tests were determined to be within the acceptable range. Based on the patient's family history and clinical and radiological characteristics, the diagnosis of Papillon-Lefèvre syndrome (PLS) was established.

Treatment

**Figure 6:**
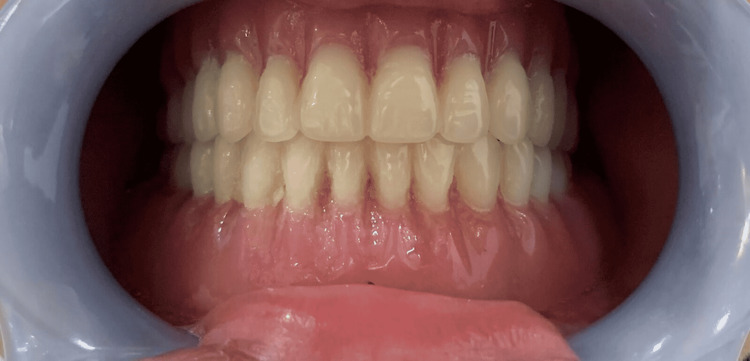
Fabrication of complete dentures

**Figure 7:**
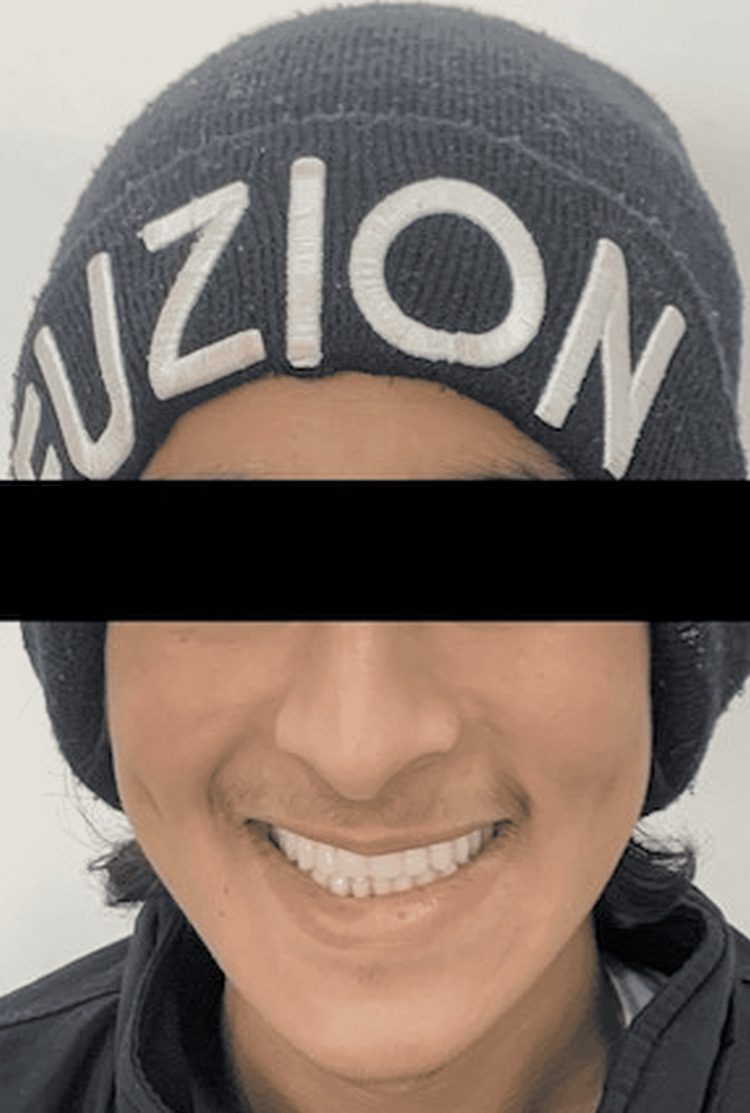
After the fabrication of complete dentures, the frontal profile of the patient with improved facial height.

Rehabilitation strategies that target both the physical and mental well-being of the patient have been examined. Maxillary and mandibular full dentures were manufactured, and facial height was enhanced after the extraction of the remaining permanent mobile teeth and the installation of dentures (Figures 6, 7). After the age of 18, dental implants may be considered.

Topical use of keratolytic drugs containing 5% salicylic acid was used to treat skin lesions. As a treatment for excessive sweating, antiperspirants and deodorants were recommended Acitretin 25 mg pill, once a day was recommended for the treatment of keratinizing diseases such as PLS, according to the guidelines. In order to minimize social stigma and psychological distress for the patient and to enhance the overall quality of life for developing children, it is necessary to identify and treat PLS as soon as possible.

## Discussion

As an X-linked condition, PLS is very uncommon. Primary and permanent teeth are lost prematurely as a result of both PPK and severe early-onset destructive periodontitis, which are the two hallmarks of the condition known as PLS. For PLS, the underlying cause is unknown. It demonstrates a complex aetiology, including genetic, immunological, and microbiological factors [12].

It is possible to determine whether a patient has PLS by a careful review of their medical and dental histories, as well as their family history. These characteristic symptoms were present in the patient in the current case, as were the clinical findings, prior family history, dental history, and radiographic results. As in AlBarrak ZM et al.'s study of five instances of PLS among siblings of the same Saudi Arabian family, consanguinity in the family might be regarded as an etiological factor in the current case with the history of the same illness identified in the sibling brother. Alleles of cathepsin C (CTSC) on chromosome 11q14-q21 seem to be affected by genetic changes between D11S4082 and D11S931 [13,14].

It has been shown that CTSC enzyme activity may be used to diagnose patients with PLS based on laboratory testing for urine analysis. Enzymatic activity is lowered by around 20% in those who are heterozygous for the mutation and carry it. In contrast, homozygous cathepsin C alleles reveal less than 10% of normal activity in the hydrolysis of synthetic substrate glycil-arginine-7-amino-4-methylcoumarin hydrolysis in the patients [15]. As homozygous for a common cathepsin C mutation, the vast majority of patients with PLS have recessive characteristics. Palmoplantar hyperkearatosis and early-onset periodontitis are not seen in family members that have the cathepsin C hetezygous mutasyns. PLS produced by a full or near-total loss of CTSC activity may display palmoplantar keratosis or merely early periodontitis. PLS should be included in a patient's differential diagnosis because of these features. The present patient's parents were both healthy and had no family history of the disease, which indicates an autosomal recessive inheritance pattern. Immunological factors have been described in the literature as contributing to PLS. HLA antigens (human leukocyte antigens), which enable the presentation of antigens to T cells during adaptive immune responses, aid in the presentation of antigens to T cells (HLAs). In polymorphonuclear neutrophils from PLS patients, serine proteinases were weak (PMNs). In the process of destroying bacteria, PMNs use serine proteases and serine proteinases. Periodontitis is more likely to happen if you don't get enough of certain nutrients [16].

Microbiological studies [17] have shown that germs in the mouths of PLS patients, such as *Actinobacillus actinomycetemcomitans*, *Porphyromonas gingivalis*, *Fusobacterium nucleatum*, and *Treponema denticola*, make periodontitis worse. Falx Cerebral malformations and a delay in the development of the soma are linked traits. Other associated symptoms and findings include pyogenic skin infections, nail dystrophy, and hyperdidrosis [18]. Previous studies reported multisystem abnormalities in a patient with PLS, aged 17 years old, male Saudi, with features such as squint, glaucoma of the right eye, pectus carinatum deformity, grade II/IV pansystolic murmur, and tappered phalanges [19], which contradicts the current study with no bone abnormalities. If caught early and treated promptly, gum disease and tooth loss may be avoided. Periodontal infections should be stopped at their source, and a safe place for permanent teeth to come in should be made. The treatment modalities should focus on factors such as restoration of function, esthetics, and psychological wellness of the patient. This requires cooperation from multi-specialties such as dermatologists, pediatricians, periodontists, and prosthodontists.

## Conclusions

The role of consanguinity in families, especially among the Arab population, is considered a key etiological factor for this intriguing syndrome, and measures should be taken to prevent such marriages within close relations. such as first cousins. It is common for people with the Papillon-Lefèvre syndrome (PLS) to be stigmatised because of their early edentulous status. Consequently, early detection of this illness is critical to improving a person's general well-being. A team of professionals working together to give the best possible care to PLS patients is helpful.
